# Patterns of Gene Conversion in Duplicated Yeast Histones Suggest Strong Selection on a Coadapted Macromolecular Complex

**DOI:** 10.1093/gbe/evv216

**Published:** 2015-11-11

**Authors:** Kathy Scienski, Justin C. Fay, Gavin C. Conant

**Affiliations:** ^1^Division of Animal Sciences, University of Missouri, Columbia; ^2^Present address: Genetics Graduate Program, Texas A&M University, College Station, TX.; ^3^Department of Genetics, Washington University; ^4^Center for Genome Sciences and Systems Biology, Washington University; ^5^Informatics Institute, University of Missouri, Columbia

**Keywords:** genome duplication, gene conversion, histones

## Abstract

We find evidence for interlocus gene conversion in five duplicated histone genes from six yeast species. The sequences of these duplicated genes, surviving from the ancient genome duplication, show phylogenetic patterns inconsistent with the well-resolved orthology relationships inferred from a likelihood model of gene loss after the genome duplication. Instead, these paralogous genes are more closely related to each other than any is to its nearest ortholog. In addition to simulations supporting gene conversion, we also present evidence for elevated rates of radical amino acid substitutions along the branches implicated in the conversion events. As these patterns are similar to those seen in ribosomal proteins that have undergone gene conversion, we speculate that in cases where duplicated genes code for proteins that are a part of tightly interacting complexes, selection may favor the fixation of gene conversion events in order to maintain high protein identities between duplicated copies.

## Introduction

Protein interactions underlie many cellular functions (Bork et al. 2004; Gavin et al. 2006; Krogan et al. 2006). Because these interactions appear to be well conserved (Qian et al. 2011), the evolution of protein complexes occurs in two dimensions, with the protein sequences of the interacting partners being under selection to maintain the interaction (Mintseris and Weng 2005; Codoner and Fares 2008; Liberles et al. 2012), while at the same time complexes can diversify or subfunctionalize through gene or genome duplications (Wagner 2001, 2003; Kunin et al. 2004; Li et al. 2006).

As it happens, there has been considerable discussion as to how the presence of one or more protein interactions alters the propensity for a gene to undergo duplication in the first place (He and Zhang 2006; Li et al. 2006; Pérez-Bercoff et al. 2010; Zhu et al. 2013). The evidence to date suggests that duplications of single genes are biased toward genes with fewer interactions than average, but a genome duplication will tend to preserve duplicates with larger numbers of interactions (Hakes et al. 2007; Pérez-Bercoff et al. 2010). This observation would be in accord with recent theory on the importance of maintaining the proper balance of genes and proteins in cells (Veitia et al. 2008). The “dosage balance hypothesis” postulates that genes whose functions involve precise interactions with other genes’ products will be under selection against large changes in dosage, hence the rarity of single gene duplications among such genes. Because whole genome duplications (WGDs) double all genes simultaneously, dosage balance would predict that highly interacting genes would instead be common among the surviving WGD duplicates, which is exactly the pattern observed (Papp et al. 2003; Edger and Pires 2009; Freeling 2009; Makino and McLysaght 2010; Birchler and Veitia 2012; Veitia et al. 2013).

Although a WGD transiently doubles the entire genome, many of the resulting duplicates are lost through “fractionation” (Sémon and Wolfe 2007; Woodhouse et al. 2010). Dosage balance is a critical force in this process, with genes coding for proteins involved in processes such as transcription or complex formation being maintained in duplicate (Blanc and Wolfe 2004; Maere et al. 2005; Carretero-Paulet and Fares 2012) to maintain a balance in dosage. Indeed, we were recently able to show that not only were genes whose encoded proteins have many interactions overretained in duplicate after WGD, but that, when they were lost, it was more likely that interacting pairs would be lost together (Conant 2014).

Although surviving duplicates may have initially been preserved because the members of a complex or group of interacting genes were under selection to remain in dosage balance, it is not expected that such selection will be active indefinitely, nor that the functions of the retained duplicates will remain constant (Conant et al. 2014). Instead, the surviving duplicates have a number of potential fates, including retained redundancy (Keane et al. 2014), functional innovation (Boles et al. 1997; Özcan and Johnston 1999; Rodríguez et al. 2001), and subfunctionalization (van Hoof 2005; Hittinger and Carroll 2007). We have previously studied a group of duplicates with apparent redundancy: The yeast ribosomal proteins (Planta and Mager 1998; Kellis et al. 2004; Kim et al. 2009), which in many cases consist of duplicate pairs with identical amino acid sequences. We found that these duplicates, created by WGD, were undergoing recurring gene conversions (GCs; Evangelisti and Conant 2010). Indeed, these conversions were so numerous that they gave rise to the mistaken impression that conversion was rampant in yeast (Gao and Innan 2004). In fact, they are the exception: GC is otherwise rare in bakers’ yeast (Casola et al. 2012).

GC occurs when one piece of DNA is “overwritten” by a second (Arnheim et al. 1980; Miyata et al. 1980; Scherer and Davis 1980). Mechanisms may include DNA/DNA-based recombination between homologous sequences (Chen et al. 2007) in mitosis or meiosis or events involving an RNA intermediate (Derr and Strathern 1993; Storici et al. 2007). It may be observed between tandemly duplicated DNA or between homologous regions on different chromosomes, as was the case for the ribosomal proteins (Scherer and Davis 1980; Chen et al. 2007).

Here we present a second example of GC among WGD-produced duplicates, namely that of histone genes. Histones have been known to undergo conversion for some time (Maxson et al. 1983; Taylor et al. 1986; Matsuo and Yamazaki 1989; DeBry and Marzluff 1994; Wang, Krasikov, et al. 1996; Wang, Tisovec, et al. 1996; Baldo et al. 1999; Liao 1999; but see Piontkivska et al. 2002; Rooney et al. 2002). The bakers’ yeast *Saccharomyces cerevisiae* has duplicate copies of the four histones whose origins predate the yeast WGD (Byrne and Wolfe 2005). These genes are somewhat diverged in sequence (ranging from *K*_s_ = 0.08, *K*_a_ ≈ 0 for the *HHF1*/*HHF2* pair to *K*_s_ = 0.49, *K*_a_ = 0.03 for *HTB1* and *HTB2*) and conversion among them was not initially suspected (Maxson et al. 1983). However, a more recent analysis suggested conversion at least between the *HHF1/2* pair (Kellis et al. 2004). The picture is more complicated in other yeast genomes. In addition to the old duplicates (where rejecting the null hypothesis of no GC is more challenging; Casola et al. 2012), these other species have histone duplicates produced by the yeast WGD itself, and, for some of those duplicates, we find clear evidence of conversion.

The existence of a second example of frequent conversion post-WGD potentially helps explain the evolutionary force underlying the fixation of these conversion events. Redundancy in duplicate genes is difficult to sustain by natural selection (Cooke et al. 1997; Nowak et al. 1997; Wagner 2000; Qian et al. 2010), but ribosomal proteins and histones may be exceptions to this rule due to the requirement for high expression of these types of genes (Kondrashov FA and Kondrashov AS 2006; Ihmels et al. 2007; Qian et al. 2010). Such selection on expression magnitude, in combination with selection to maintain dosage balance, would explain the survival of the WGD duplicates for these two classes of genes. GC, then, would have the secondary role of keeping the sequences of the duplicates similar enough that both copies function equally well in the ribosome and the nucleosome, both of which are tightly interacting and essential complexes.

## Methods

### Data Collection

Gene sequences from 8 histones (2 ancient duplicates each of histones 2A, 2B, 3, and 4) from 12 post-WGD yeasts were obtained from YGOB (Yeast Genome Order Browser Project; Byrne and Wolfe 2005). A histone pair was categorized as having undergone conversion if the two WGD-produced paralogs from a species had higher protein sequence identity to each other than either did to any homolog in its nearest relative. Histone genes *HTB2*, *HHT1*, and *HHT2* were not found to have conversion events and were not further analyzed. The remaining five genes (table 1) had duplicates with evidence of conversion in one or more yeasts.

**Table 1:** Patterns of Histone Protein Sequence Identity and Gene Phylogenies Provide Evidence for Gene Conversion among Duplicated Histones

*Saccharomyces cerevisiae* gene^a^	Gene type	Gene IDs	Dist(*D*_1_,*D*_2_)^b^	Min[Dist(*D*_1_,*O*),Dist(*D_2_*,*O*)]^c^	ln*L*_spp_^d^	ln*L*_GC_^d^	ln*L*_PhyML_^d^
*HTA1*	*D*_1_^e^	TPHA0L01110	0.008	0.038	−1,586	−1,534	−1,524
	*D*_2_^e^	TPHA0C02050					
	*O*^e^	Kpol_1031.53					
*HTA2*	*D*_1_^e^	KAFR0C00780	0.0	0.015	−1,357	−1,324	−1,316
	*D*_2_^e^	KAFR0F02490					
	*O*^e^	KNAG0K01430					
*HTB2*	*D*_1_^e^	KAFR0C00770	0.030	0.091	−1,294	−1,259	−1,258
	*D*_2_^e^	KAFR0F02480					
	*O*^e^	KNAG0K01420					
*HHF1*	*D*_1_^e^	KAFR0C00700	0.0	0.010	−1,251	−1,174	−1,146
	*D*_2_^e^	KAFR0A01280					
	*O*^e^	KNAG0J01060					
	*D*_1_^e^	CAGL0C04136g	0.0	0.010			
	*D*_2_^e^	CAGL0H09834g					
	*O*^e^	YBR009C (*HHF1*)					
*HHF2*	*D*_1_^e^	NDAI0B03480	0.0	0.010	−1,011	−963	−962
	*D*_2_^e^	NDAI0G00750					
	*O*^e^	NCAS0B06180					
	*D*_1_^e^	NCAS0B06180	0.0	0.010			
	*D*_2_^e^	NCAS0G03710					
	*O*^e^	NDAI0B03480					

^a^*Saccharomyces cerevisiae* histone gene name. Note that *S. cerevisiae* has no surviving histone duplicates from the WGD, making these names unambiguous.

^b^Proportion of amino acid difference between the two paralogs (*D*_1_ and *D*_2_) created by WGD.

^c^Minimum of the proportion of amino acid difference between one of the two orthologs (*D*_1_ or *D*_2_) and the nearest homolog in its nearest species relative (*O*).

^d^ln-likelihood of the full sequence alignment fit to the assumed species tree (ln*L*_SPP_), the gene conversion tree (ln*L*_GC_), or the phylogeny estimated by PhyML (ln*L*_PhyML_). See Methods for details.

^e^Relationship between two paralogs hypothesized to have undergone gene conversion (*D*_1_ and *D*_2_) and an assumed ortholog of *D*_1_, *O* (see table 2 for precise orthology inferences).

### Orthology Inference Using Polyploidy Orthology Inference Tool to Establish Expected Gene Relationships

Recall that we have here the special case of GCs after WGD. Thus, paralogs produced by WGD should be more distantly related to each other than to their orthologs in other genomes sharing the WGD. If two WGD-produced paralogs are found to be more closely related to each other than either is to its respective ortholog in another post-WGD yeast, that is evidence of GC. We used POInT (Polyploidy Orthology Inference Tool) to estimate, for each of the potentially converted histone genes, its ortholog in its nearest neighboring genome. If one genome was missing both copies of that histone, we removed that species from our orthology inferences.

### Triplet Tests for Gene Conversion

There are several signatures that can be used to infer GC events. For arbitrary sequences, the GENECONV program (Sawyer 1989) identifies runs of sequence similarity between pairs of sequences that are unexpectedly long given the overall distribution of similar bases in a sequence alignment, while controlling for the structure of the genetic code. The structure of this computation illustrates some of the difficulties in testing for GC. Such conversion events violate two key assumptions of the standard models of molecular evolution. First, a GC event can result in local regions of a sequence that do not follow either the overall gene tree for that gene or the species tree. More seriously, GC events, if they result in a “track” of converted bases, also violate the assumption of independence of sites in an alignment.

These difficulties make it difficult to explicitly account for GC in evolutionary models. Instead, using the WGD as our baseline, we have chosen to test for GC by seeking to reject a null model that does not include such events. As we did in our previous analyses of ribosomal proteins (Evangelisti and Conant 2010), we used a triplet-based test to compare two duplicated histone genes (*D*_1_ and *D*_2_) to the nearest ortholog (*O*) of *D*_1_, identified using POInT. Note that *D*_1_ and *O* are expected to be phylogenetically much closer (separated by a recent speciation) than are *D*_1_ and *D*_2_ (which last shared a common ancestor at the WGD). We first aligned the three sequences using T-Coffee (Notredame et al. 2000). For each of the three branches of the tree (*D*_1_, *D*_2_, and O), we made maximum-likelihood estimates for the number of nonsynonymous (*K*_a_) and synonymous (*K*_s_) substitutions per site (Conant and Wagner 2003). Using a likelihood-ratio test, we then assessed the statistical support for an inference of GC between genes *D*_1_ and *D*_2_ (Sokal and Rohlf 1995). We calculated the likelihood of the alignment allowing the three values of *K*_a_ (or *K*_s_) to be independent. We compared twice the difference in that ln-likelihood with that of a model where the *K*_a_ (or *K*_s_) leading to D_1_ was constrained to be no less than that leading to *O* using a chi-square distribution with 1 degree of freedom (table 2).

**Table 2:** Triplet-based Relative Rate Tests Coupled to Orthology Predictions Show Evidence for Gene Conversion at Synonymous Sites of Duplicated Histones

*Saccharomyces cerevisiae* gene^a^	Gene type	Species-specific genes	Probabilities of orthology relationship^b^	*K*_a_^c^	*P*^d^	*K*_s_^c^	*P*^d^
*HTA1*	*D*_1_^e^	TPHA0L01110	>0.99	0.004	0.15	0.062	**0.016**
	*D*_2_^e^	TPHA0C02050		≈0		0.039	
	*O*^e^	Kpol_1031.53		0.015		0.186	
*HTA2*	D_1_^e^	KAFR0C00780	>0.99	≈0	**=0.02**	≈0	**<0.001**
	D_2_^e^	KAFR0F02490		≈0		0.189	
	O^e^	KNAG0K01430		0.012		0.551	
*HTB2*	*D*_1_^e^	KAFR0C00770	>0.99	0.008	0.08	0.156	**0.011**
	*D*_2_^e^	KAFR0F02480		0.015		0.098	
	*O*^e^	KNAG0K01420		0.027		0.411	
*HHF1*	*D*_1_^e^	KAFR0C00700	0.97	≈0	0.07	0.011	**<0.001**
	*D*_2_^e^	KAFR0A01280		≈0		0.124	
	*O*^e^	KNAG0J01060		0.010		0.459	
	D_1_^e^	CAGL0C04136g	=0.97	≈0	=0.24	0.028	**<0.001**
	D_2_^e^	CAGL0H09834g		≈0		0.001	
	O^e^	YBR009C (*HHF1*)		0.005		0.426	
*HHF2*	*D*_1_^e^	NDAI0B03480	>0.99	≈0	0.20	0.054	**<0.001**
	*D*_2_^e^	NDAI0G00750		≈0		0.076	
	*O*^e^	NCAS0B06180		0.004		0.315	
	*D*_1_^e^	NCAS0B06180	>0.99	≈0	0.21	0.071	**0.005**
	*D*_2_^e^	NCAS0G03710		≈0		0.088	
	*O*^e^	NCAS0B06180		0.004		0.297	

^a^*Saccharomyces cerevisiae* histone gene name (see table 1).

^b^Estimated probability of the full set of orthology relationships used for this and later analyses from POInT. Thus, of all possibe orthology relationship, what proportion of the probability is apportioned to the one described.

^c^Using our triplet-based likelihood approach (Conant and Wagner 2003), we estimated for each of the three branches (corresponding to the three genes) the number of nonsynonymous (*K*_a_) and synonymous (*K*_s_) substitutions per site.

^d^*P* value for the hypothesis test of equal values of *K*_a_ (or *K*_s_) for *D*_1_ and *O*. This condition corresponds to the hypothesis of no gene conversion: *D*_1_ and its ortholog *O* are equally distant from paralog *D*_2_. The test is based on a likelihood-ratio test of a null model where all values of *K*_a_ (or *K*_s_) are free to an alternative model where the *K*_a_ (or *K*_s_) values of *D*_1_ and *O* are forced to be equal. The *P* value was computed by comparing twice the difference in ln-likelihood to a chi-square distribution with one degree of freedom. Values shown in bold are significant at *P*<=0.05.

^e^Relationship between two paralogs hypothesized to have undergone gene conversion (*D*_1_ and *D*_2_) and the orthology of *D*_1_, *O*.

### Gene Tree Tests of Conversion

**Fig. 1.—:**
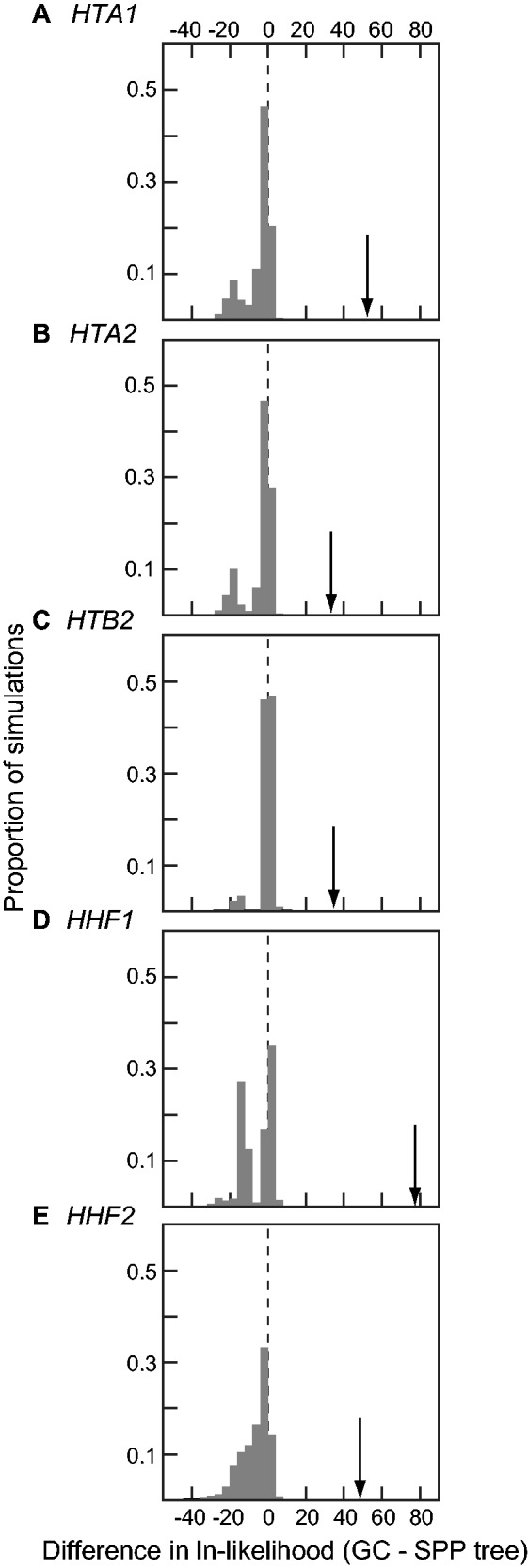
For all five examples of post-WGD GC, a tree joining the putatively gene-converted ohnologs explain the sequence data better than does the post-WGD species phylogeny. For each of the 5 loci, we simulated 1,000 sequence alignments under the presumed species tree (SPP) of figure 2 (omitting any branches where gene loss had occurred). We then analyzed those alignments under both the SPP tree and all possible GC trees. We calculated the difference in ln-likelihood between the best GC tree and the SPP tree. Thus, values greater than zero implies that the GC tree better explains the data than does the SPP tree. The proportion of simulations with a given value of the difference in ln-likelihood for the two trees is shown on the *y*-axis. For reference, we show the improvement in ln-likelihood seen under the GC tree for the real data with arrows.

We analyzed alignments of all post-WGD histones for each of the five genes showing evidence of conversion. After T-Coffee alignment, we estimated maximum-likelihood gene trees for the alignments with PhyML (Guindon and Gascuel 2003). Using POInT and the species tree topology from YGOB (Byrne and Wolfe 2005), we created an expected species tree for the histone genes and putative conversion trees as described in the Results section. For all three trees (PhyML estimate, species tree, and converted tree) we estimated the likelihood of the alignment using the codon model of Muse&Gaut/Goldman&Yang (MY/GY; Goldman and Yang 1994; Muse and Gaut 1994). Because the species and GC trees are not nested within each other, we cannot use the chi-square approximation to describe the differences in ln-likelihood between these two trees. Instead, we used sequence simulations to assess if the GC tree provides a better fit to the alignments than does the species tree. Using our own simulation package, we simulated sequences of the same length on the species tree, using that tree’s corresponding inferences of the parameters of the MY/GY model. We then computed the ln-likelihood of these simulated alignments on both the species tree and all possible GC trees, retaining the GC with the largest ln-likelihood. The distributions of differences between this ln-likelihood and that of the species tree for the 1,000 simulations are illustrated in figure 1.

## Results

GC can produce, in an ancient shared gene duplication, a situation where two paralogs from one species are more similar to each other in sequence than either is to its ortholog in the other species. Unfortunately, such a pattern of sequence similarity could also result from independent gene duplications in the two lineages. As we have previously discussed, the special case of a genome duplication allows us to avoid this confound, because the independent signal of shared gene order, or synteny, allows us to infer orthology between duplicated genes regardless of patterns of sequence evolution (Casola et al. 2012).

**Fig. 2.—:**
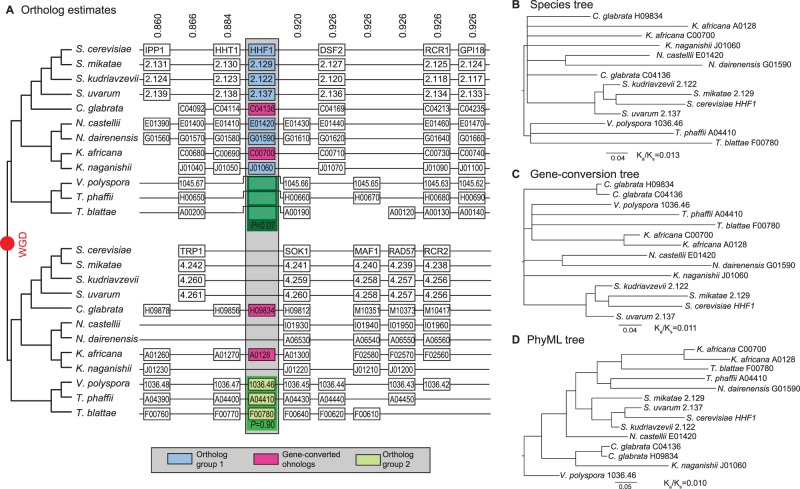
(*A*) Orthology prediction for 12 post-WGD yeasts from POInT for the genomic region around histone 4 (*HHF1*). WGD produced two duplicated regions, shown as the top and bottom panels. For this set of genes (gray column) there are two orthology assignments of reasonably high probability: One that makes the genes from *Vanderwaltozyma polyspora*, *Tetrapisispora phaffii*, and *Tetrapisispora blattae* paralogous to the nine genes in the upper panel (*P* = 0.90) and one that makes them orthologous (*P = *0.07). Importantly, neither of these relationships contradicts the inference that gene H09834 from *Candida glabrata* and gene A0128 from *Kazachstania africana* are paralogous to the upper group of nine genes (hence *P > *0.97 for that assignment). As a result, we expect the gene tree of these 11 sequences to have these 2 genes cluster outside of the other 9, as depicted in the species tree of *B*. Instead, the two genes in pink from *C. glabrata* and *K. africana* are each other’s closest relatives in the tree, a result only explicable under the hypothesis of gene conversion. (*B*) Fit of the *HHF1* sequence alignment to the species tree from *A* under the MG/GY 94 model. (*C*) Fit of the *HHF1* sequence alignment to a hypothesized gene conversion tree under the MG/GY 94 model. (*D*) Maximum-likelihood estimate of the gene tree from PhyML (see Methods) for *HHF1* fit to the MG/GY 94 model.

Here, we have applied a synteny-based approach to the analysis of several WGD-produced histone duplicates that are identical or nearly identical at the amino acid level. Importantly, these paralogs show lower identity to the most closely related of their homologs in their nearest relative than to each other (fig. 2 and table 1). However, for a strict test of GC, we need to compare each paralog with its ortholog in its nearest relative. (The full list of duplicated histone genes in these 12 taxa is available as supplementary data, Supplementary Material online.) Unfortunately, although identifying paralogs shared from the WGD in pairs of yeast genomes is now relatively straight forward (Byrne and Wolfe 2005), assigning orthology between those genes is more difficult (fig. 2*A*). We have developed a software tool called POInT that uses synteny (Gordon et al. 2009) and a maximum-likelihood phylogenetic model of gene loss to make probabilistic estimates of which homologous genes in two species sharing a WGD are actually orthologs (Conant and Wolfe 2008; Conant 2014). This program’s inferences are illustrated in figure 2*A*: The numbers above each column give POInT’s estimate of the probability of the orthology relationship shown as compared with all other 2*^n^*^−^^1^−1 possible assignments. Here *n* is the number of genomes: Each duplicated gene in a particular genome could be assigned as the ortholog of one of the two genes (or potentially a position where a gene was inferred to be lost—gaps in fig. 2*A*) in each of the other *n*−1 genomes. The synteny data strongly support the hypothesis that these histone duplicates last shared a common ancestor at the WGD event and not after the more recent speciation event. Thus, for *HTA2*, even though the amino acid sequences of the two duplicates from *Kazachstania africana* are identical, one of those two genomic loci last shared a common ancestor with its *Kazachstania naganishii* ortholog more recently than with its WGD-produced paralog. This pattern of sequence identity can either be explained by random fixations giving rise to a misleading gene tree when comparing the gene sequences (akin to lineage sorting) or by GC acting on those sequences.

We adopted two tests to choose between these hypotheses. First, as in our previous analysis, we compared the sequence divergence of the putatively converted genes with one of their orthologs using a triplet-based analysis of asymmetric evolution (Conant and Wagner 2003; Evangelisti and Conant 2010). With this approach, we compute branch-specific values for the nonsynonymous (*K*_a_) or synonymous (*K*_s_) divergence for three genes: Two WGD-produced paralogs *D*_1_ and *D*_2_ and *O*, the ortholog of *D*_1_ (table 2). If GC has not occurred, the sum of the *K*_a_ values for *O* and *D*_1_ should be less than the sum for *D*_1_ and *D*_2_. Our observation of GC implies instead that the sum of the *K*_a_ values for *D*_1_ and *D*_2_ is less than that for *D*_2_ and *O*. Thus, rejecting the null hypothesis that *K*_a_ for *D*_1_ is equal to that for *O* is equivalent to rejecting the null hypothesis of no GC (see Methods). We suspected that the high amino acid identities of the histones would make this test relatively insensitive. And, indeed, as table 2 shows, we were unable to reject the hypothesis of equal divergence in *K*_a_ between *D*_1_ and *O* in six of the seven cases. However, in all cases we saw significant evidence of conversion in *K*_s_ values (*P* ≤ 0.015, likelihood-ratio test; Methods).

Because this first test was inconclusive, we next placed the putative conversion events onto the species phylogeny postulated by YGOB (Byrne and Wolfe 2005; Gordon et al. 2009). For the five putative cases of conversion in table 1, we assigned orthology for the duplicated genes using our orthology inference tool POInT (see Methods). The ln-likelihood of the species phylogeny for each alignment was compared with all possible GC-type gene trees (table 1), using a codon model of evolution (see Methods). These trees were created by taking the species phylogeny and moving one of the putatively gene converted sequences to be sister to its WGD-produced paralog (fig. 2). From this set of possible rearranged trees, we retained the one with the highest ln-likelihood. In all cases, this best GC tree had a higher ln-likelihood than the species tree. To assess if this higher ln-likelihood was statistically significant, we simulated 1,000 alignments on the species tree under the same codon model. For each simulation, we then compared the ln-likelihood of the species tree with that of the GC tree found to have the highest likelihood for that simulation (fig. 1). In no case did the simulated data sets have an improvement ln-likelihood from the optimal GC tree as large as the improvement seen in the real data (*P < *0.001; dashed line in fig. 1). We therefore conclude that these sequences show significant evidence for GC, as the only difference between the species tree and the GC trees is the position of the putatively converted paralogs.

### Unusual Substitution Patterns among Histone Genes

**Fig. 3.—:**
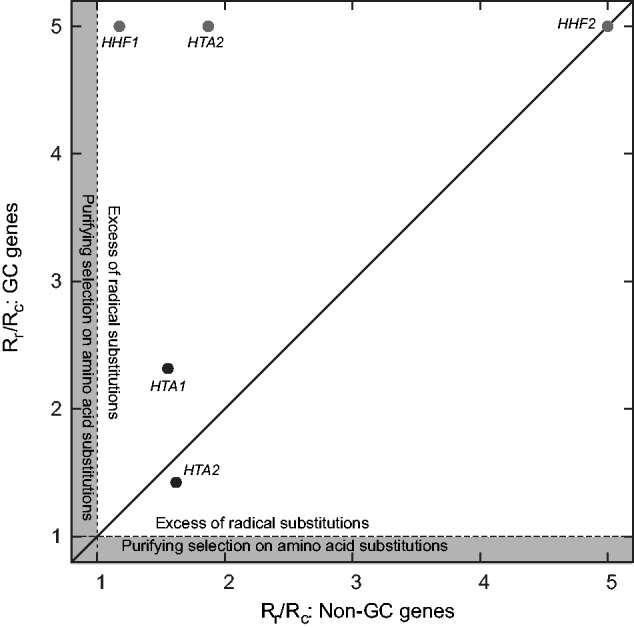
An excess of radical amino acid substitutions is observed among the histones of the post-WGD yeasts, a trend that is most marked among the clades having undergone gene conversion. On the *x-*axis is the ratio of the rate of radical (*R*_r_) to conservative (*R*_c_) substitutions along all branches of the phylogeny not showing evidence of gene conversion (as estimated from our ML code, see Methods). The gray area indicates the realm of purifying selection (*R*_r_/*R*_c_ ≤ 1.0). On the *y-*axis is the same statistic for the three branches showing gene conversion (e.g., the two gene converted tips and their shared ancestral branch). The line *y = x* indicates equal values of *R*_r_/*R*_c_ for the two sets of branches. Points in gray with a value of 5.0 have *R*_c_ = 0 (and hence an actual ratio that is undefined).

To further explore the evolution of these converted genes, we applied an SG (Similarity Groups) model (Conant et al. 2007) to each histone alignment used above. The SG model separates the amino acids into polar and nonpolar residues and allows one selective constraint (*R*_c_) for substitutions within the same group and a second (*R*_r_) for substitutions between groups. The parameter estimates for the MG/GY and SG models are given in supplementary table S1, Supplementary Material online. The supplementary figure, Supplementary Material online, illustrates the location of substitutions in the converted histone genes relative to the *S. cerevisiae* structural model (White et al. 2001), while supplementary table S2, Supplementary Material online, gives the locations of all substitutions relative to the crystal structure. In general, most genes show *R*_r_ < *R*_c_, because substitutions that do not change polarity should be less drastic and hence less likely to provoke purifying selection (Zhang 2000). Unexpectedly, however, all the histone genes with at least one instance of conversion showed *R*_r_ > *R*_c_ (fig. 3). Even in sequences that are simply drifting, observing *R*_r_ > *R*_c_ in five of the five cases is unexpected (i.e., under the null hypothesis that *R*_r_ exceeds *R*_c_ in 50% of the cases; *P = *0.03, binomial test). However, after a false-discovery rate correction, we cannot reject the null hypothesis of *R*_r_ = *R*_c_ for any of those five alignments (*P* > 0.05, likelihood-ratio test with FDR correction; Benjamini and Hochberg 1995). Note however that, in three cases, the genes inferred to have undergone GC showed at least one radical amino acid substitution and no conservative substitutions (fig. 3). These patterns are again unexpected if only strong purifying selection is acting on histone genes and may suggest the presence of coevolution among histones.

## Discussion

With a second group of genes that have undergone GC since their most recent speciation event, we may have the beginnings of a pattern. In particular, the two groups of genes share two important features: They are well conserved, and they function as part of essential macromolecular complexes. The dosage balance hypothesis predicts that such genes are likely to be retained in duplicate after the WGD due to their many interactions (Veitia et al. 2008). But why might GC occur? We speculate that the tight nature of the complexes may be the reason. The fixation of a GC might be beneficial in the presence of coevolution between a pair of duplicates and their interaction partner(s). If a change in the interaction partner has resulted in a compensating mutation in one of the duplicates, the transfer of that change to the second duplicate by GC would then be beneficial because it allows the complex to be maintained no matter the duplicate copy used. This idea is speculative because we do not yet have a test that would indicate whether a gene converting mutation became fixed through selection. However, there are several suggestive points. Among the ribosomal proteins, the signal of conversion was much stronger for nonsynonymous substitutions than for synonymous ones, a fact difficult to reconcile with drift. Here, although we did not find such a difference in the two types of substitution, we do note that the duplicated genes are not identical at the nucleotide level despite showing a signal of conversion in synonymous substitutions (table 2). Similarly, the amino acid substitutions that have occurred actually appear biased toward more radical amino acid changes (fig. 3). However, the observation of *R*_r_ > *R*_c_ should not be taken as “classical” positive selection in this instance and not merely because the statistical evidence for rejecting *R*_r_ = R_c_ is lacking. (As an aside, we note that while the use of *R*_r_ and *R*_c_ has been criticized, our likelihood-based estimates of these parameters do not suffer from many of the biases seen with older estimation methods; Dagan et al. 2002.) The reason for caution in interpreting these values is that, in these histone genes, there are forces at work beyond those of mutation and selection that form the basis of the standard models. In addition to the mutational process generating variants in the duplicate copies, there is a GC process that may either copy a variant into the other duplicate or eliminate it through the alterative conversion. Selection in turn operates on both types of event, while at the same time there is a potential for selection driving coevolution between interacting partners in the nucleosome. With all these forces at work, our intuition as to how evolution is operating is likely to be rather poor: We simply argue that our observations here are not consistent with drift or the simplest form of purifying selection.

We also note that, because GC often occurs in tracks of multiple bases, the observation of conversion at synonymous positions might then be a hitchhiking effect of the selective preservation of nonsynonymous conversions. We also previously showed with the ribosomal proteins that expression level alone was not sufficient to explain the frequency of GC in these genes, despite the existence of RNA-based GC mechanisms in yeast (Derr and Strathern 1993; Storici et al. 2007).

These results also fit into the larger picture of the yeast genome duplication. An elegant analysis by Marcet-Houben and Gabaldon (2015) used gene trees to infer that the yeast WGD was very likely an allopolyploid event, e.g., the merging of the “diploid” genomes of two distinct species. Interestingly, however, the extant duplicated genomes are not an equal mix of these two source genomes: One genome seems to have come to dominance, probably by a combination of biased gene losses and GCs (Tang et al. 2012; Wolfe 2015). We speculate that the process of GC observed here for the histone and ribosomal protein genes may have started with selection acting for this sort of genome dominance. In that view, cassettes of critical genes, such as those for histones, ribosomal proteins, or DNA repair enzymes, would have come from both parents, but might not function interchangeably. Indeed, such a combination might set the stage for dominant negative interactions, where the presence of an alternative version of a particular gene caused a reduction in fitness (De Smet et al. 2013). We previously observed one potential solution to this conundrum: The duplicate copies of genes for mitochondrially targeted proteins and DNA repair enzymes were rapidly lost after the yeast WGD (Conant 2014). GC is a second solution, where two genetic loci are retained but both contain sequences originating from a single parent.

Duplicate genes continue to surprise us with their multifaceted evolutionary patterns (Hahn 2009). In so doing, they justify Ohno’s interest not only from an evolutionary perspective but also because the response of a biological complex to the duplication of its members reveals a good deal about its function.

## Supplementary Material

Supplementary figure, data, tables S1 and S2 are available at *Genome Biology and Evolution* online (http://www.gbe.oxfordjournals.org/).

Supplementary Data
