# Supplementary material for: Patterns of Gene Conversion in Duplicated Yeast Histones Suggest Strong Selection on a Coadapted Macromolecular Complex
Source: Genome Biol Evol. 2015 Nov 11;7(12):3249–58. doi: 10.1093/gbe/evv216 (PMC4700949; doi:10.1093/gbe/evv216)
Supplement: Supplementary Data [file supp_7_12_3249__index.html]

Patterns of gene conversion in duplicated yeast histones suggests strong selection on a co-adapted macromolecular complex — Patterns of Gene Conversion in Duplicated Yeast Histones Suggest Strong Selection on a Coadapted Macromolecular Complex — Supplementary Data 

# Patterns of Gene Conversion in Duplicated Yeast Histones Suggest Strong Selection on a Coadapted Macromolecular Complex

## Supplementary Data

files

- Supplementary Data - pdf file
- Supplementary Data - xlsx file
- Supplementary Data - pdf file
