# Supplementary material for: Patterns of Gene Conversion in Duplicated Yeast Histones Suggest Strong Selection on a Coadapted Macromolecular Complex
Source: Genome Biol Evol. 2015 Nov 11;7(12):3249–58. doi: 10.1093/gbe/evv216 (PMC4700949; doi:10.1093/gbe/evv216)
Supplement: Supplementary Data [file supp_evv216_Supplemental_Figure.pdf]

Supplemental Figure: Locations of substitutions in gene converted histone genes relative to the *S. cerevisiae* nucleosome crystal structure.

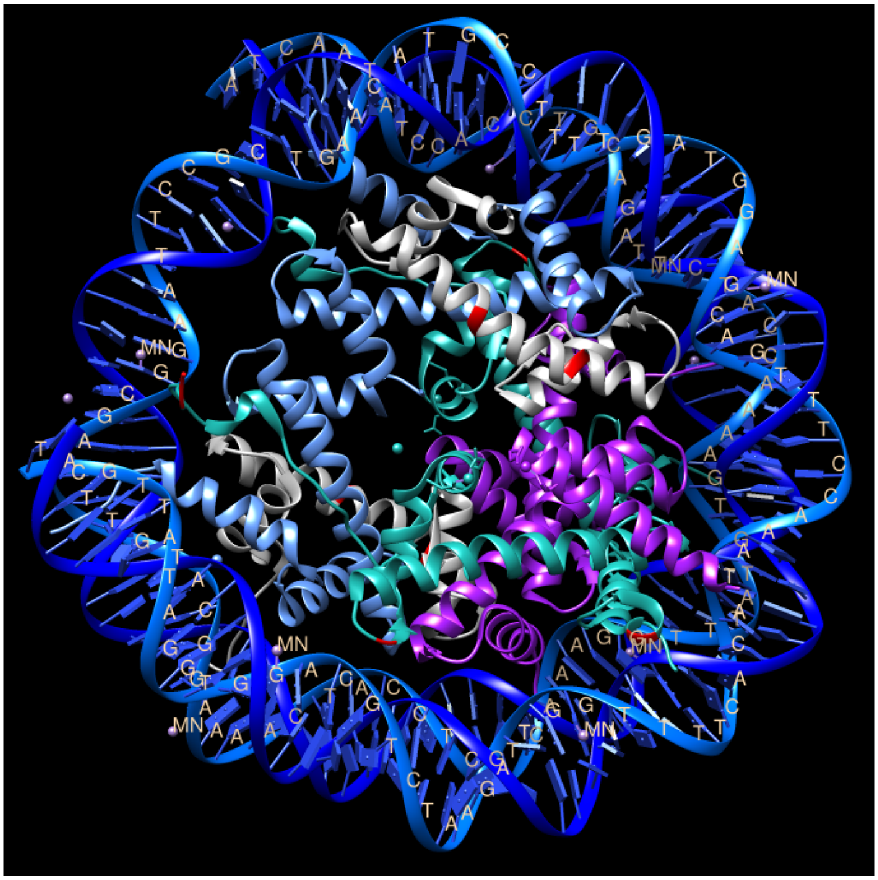

Substitutions in converted genes relative the *S. cerevisiae* nucleosome crystal structure (White, Suto, and Luger 2001) for *HTA* and *HHF* (PDB structure accession 1ID3). Cases where one or more of the converted genes differs from the sequence from which the structure is derived are shown in red. Two differences are seen in HHF (gray chain) and four in HTA (aqua). An additional nine substitutions were seen in the N-terminal portion of HTB, but that region of the sequence is not part of this crystal structure. The full list of changes, including those not in the structure, is given in Supplemental Table 2.
