# Supplementary material for: Patterns of Gene Conversion in Duplicated Yeast Histones Suggest Strong Selection on a Coadapted Macromolecular Complex
Source: Genome Biol Evol. 2015 Nov 11;7(12):3249–58. doi: 10.1093/gbe/evv216 (PMC4700949; doi:10.1093/gbe/evv216)
Supplement: Supplementary Data [file supp_evv216_Supplemental_Tables.pdf]

Supplemental Table 1: Parameter estimates for MG/GY and SG models on optimal GC trees.

***S. cerevisiae* histone gene**	**MG/GY lnL[a]**	**K_a/K_s[b]**	**SG lnL[c]**	**R_C/K_s[d]**	**R_R/K_s[e]**
HTA1	-1534.4549	0.0368611	-1533.5481	0.02975	0.05362
HTA2	-1324.1847	0.0240684	-1323.4473	0.01724	0.04044
HTB2	-1259.9214	0.0436161	-1259.412	0.03545	0.05673
HHF1	-1174.4218	0.0110795	-1173.3036	0.00706	0.0253
HHF2	-963.2829	0.0104231	-960.3585	0	0.05

a: ln-likelihood of the best GC tree under the MG/GY model (see main text). See also Table 1.

b: Estimate of the global value of K_a/K_s from the MG/GY model on the best GC tree.

c: ln-likelihood of the best GC tree under the SG (Similarity Groups) model (see main text).

d: Estimate of the rate of conservative (R_C) amino acid substitutions relative to the synonymous substitution rate for the SG model.

e: Estimate of the rate of radical (R_R) amino acid substitutions relative to the synonymous substitution rate for the SG model.

Supplemental Table 2: Locations of substitutions in converted histone duplicates.

Gene	Converted gene pair	Position[a]	*S. cerevisiae* AA[b]	Duplicate 1 AA	Duplicate 2 AA
HTA1	TPHA0L01110/ TPHA0C02050	20	A	S	S
		77	T	S	S
		125	T	V	A
HTA2	KAFR0F02490/ KAFR0C00780	124	A	P	P
		125	T	G	G
HTB2	KAFR0F02480/ KAFR0C00770	2	S	S	A
		3	A	A	K
		24	S	A	A
		25	T	S	T
		26	S	G	G
		28	D	E	E
		29	G	A	G
		33	S	T	T
		35	V	A	A
HHF1	CAGL0C04136g/ CAGL0H09834g	69	S	A	A
HHF1	KAFR0C00700/ KAFR0A01280	60	S	Q	Q
		69	S	A	A
HHF2	NCAS0B06180/ NCAS0G03710	69	S	A	A
HHF2	NDAI0G00750/ NDAI0B03480	60	S	T	T
		69	S	A	A

a: Position of the residue in the *S. cerevisiae* crystal structure.

b: Residue at that position in the structure.
